# Neutrophil-to-Lymphocyte Ratio Is Associated with Impaired Interferon-Gamma Release to Phytohemagglutinin

**DOI:** 10.1371/journal.pone.0125794

**Published:** 2015-05-11

**Authors:** Kwang-Sook Woo, Byoung-Gwon Kim, Jae-Lim Choi, Bo-Ram Kim, Kyeong-Hee Kim

**Affiliations:** 1 Department of Laboratory Medicine, Dong-A University College of Medicine, Busan, Republic of Korea; 2 Department of Preventive Medicine, Dong-A University College of Medicine, Busan, Republic of Korea; Zhongshan Hospital Fudan University, CHINA

## Abstract

**Objective:**

The neutrophil-to-lymphocyte ratio (NLR) has been shown to predict adverse outcomes in several pathologic conditions. The majority of indeterminate interferon (IFN)-γ release assays were due to inadequate IFN-γ response to the phytohemagglutinin. We sought to study the value of NLR to predict an indeterminate result of QuantiFERON-TB Gold In-Tube (QFT-GIT) performed in routine laboratory practice.

**Methods:**

Results from 2,773 QFT-GIT assays were analyzed. Data collection included demographic data, the level of IFN-γ to nil, mitogen, and TB antigen of QFT-GIT, total WBC, and a differential count. We calculated the absolute neutrophil count, lymphocyte count, and NLR.

**Results:**

Of the total, 224 (8.1%) indeterminate results were observed. Twelve (1.8%) showed indeterminate results in the NLR range from 1.71 to 2.84, but 132 (19.2%) had indeterminate results in NLR≥5.18 (p<0.0001). The likelihood ratio for indeterminate results were 2.70 (95% CI, 2.36-3.08) in NLR ≥5.18 and 1.93 (95% CI, 1.64-2.27) in lymphocyte count ≤1050/μL. NLR and neutrophil count were independent predictors for indeterminate QFT-GIT result in multiple regression analysis. The IFN-γ response to PHA was negatively associated with NLR (r=-0.33, p<0.001).

**Conclusion:**

We showed that the NLR is an independent predictor of indeterminate QFT-GIT result. Low frequency of indeterminate results in group with normal NLR may imply the importance of a balance between two cellular compartments in physiological and pathological conditions.

## Introduction

The neutrophil-to-lymphocyte ratio (NLR) is a simple ratio of the absolute neutrophil and lymphocyte counts obtained on the differential section of the total white blood cell count (WBC) of a complete blood cell (CBC) count. NLR is a marker of inflammatory response and has been shown to be associated with poor outcomes in patients with several types of disease. A high NLR was associated with an adverse overall survival in many solid tumors in a systematic review [1], and the NLR had independent prognostic value in various clinical utilities for patients with cancer [2]. In the cardiovascular system, the NLR has correlated with worse results in patients with acute coronary syndrome and coronary heart disease [3–5] and can independently predict coronary heart disease in an asymptomatic general population cohort [6]. Additionally, the NLR has shown to be a useful marker in kidney transplantation [7], schizopherenia [8], bacterial pneumonia [9], and Alzheimer's disease [10].

The interferon-gamma (IFN-γ) release assay (IGRA) was developed after it was recognized that IFN-γ plays a critical role in regulating cell-mediated immune responses to *Mycobacterium tuberculosis* infection. The IGRA detects sensitization to *M*. *tuberculosis* by an enzyme-linked immunosorbent assay to measure the amount of IFN-γ released in response to antigens representing *M*. *tuberculosis* compared with controls. In 2007, the QuantiFERON-TB Gold In-Tube (QFT-GIT) (Cellestis, Carnegie, Australia) became the third IGRA to be approved by the Food and Drug Administration (FDA) as an aid for diagnosing *M*. *tuberculosis* infection and has improved the limitations of a previous version of IGRA [11]. Interpretation criteria for QFT-GIT approved by the FDA were positive, negative, and indeterminate. The indeterminate interpretation has indicated an uncertain likelihood of *M*. *tuberculosis* infection.

Indeterminate response has been shown in both a low response to the phytohemagglutinin (PHA) as a mitogen (<0.5 IU/mL) and high IFN-γ response to the no antigen (nil) (>8.0 IU/mL). The rate of indeterminate response in QFT-GIT has differed depending on the disease group and has ranged from 5.8% to 10% [12–15]. The predictive factor of indeterminate response could be divided into the patient’s side and technical side. In technical aspect, prolonged delay of incubation, a manual ELISA system has been associated with indeterminate IGRA [12,16,17]. Immunosuppressive drug therapy, underlying diseases, hypoalbuminemia, low hemoglobin level and lymphocytopenia may be the risk factors of indeterminate IGRA results [12–15]. Most indeterminate QFT-GIT results were caused by inadequate IFN-γ response in the PHA and a higher risk of progression to AIDS or death was reported in advanced quantitative CD4^+^ T cell depleted HIV-1-infected patients with an indeterminate QFT-GIT result at baseline. Thus indeterminate QFT-GIT response might indicate an additional loss of global T cell function [15].

Lymphocytopenia has been a well-recognized predictor of indeterminate IGRA results [18,19]. Decreased lymphocyte count often results in high NLR. However, there is a lack of data regarding the association of NLR and the indeterminate response of IGRA. We sought to study the value of NLR to predict an inadequate IFN-γ response to PHA of the QFT-GIT performed in routine laboratory practice.

## Materials and Methods

### Ethics statement

This study was approved by the Institutional Review Board at Dong-A University Hospital (Busan, Republic of Korea). As this study was a retrospective analysis based on routine laboratory results and the data were analyzed anonymously, informed consent was waived by the Institutional Review Board.

### Study subjects

From March 6, 2009 to December 27, 2012, 3183 tests consecutively referred by clinicians to the serology laboratory in Dong-A University Hospital for a QFT-GIT were initially enrolled in this study. The indication for the QFT-GIT was as follows: tuberculosis disease, contact investigation, tumor necrosis factor-blocker use, and suspected symptoms for tuberculosis disease. We collected data of the CBC count requested on the same day of the QFT-GIT. Data collection included demographic data, the level of IFN-γ to nil, mitogen, and TB antigen of QFT-GIT, total WBC, and differential count. We calculated the absolute neutrophil count, lymphocyte count, and NLR. The CBC count was performed by a Sysmex XE2100 hematology analyzer (Kobe, Japan).

### QFT-GIT assay

The QFT-GIT was carried out according to the manufacturer’s instructions [20]. A 1mL sample of blood was collected directly into the manufacturer-provided nil, TB antigen, and mitogen tubes and was shaken 10 times to ensure proper mixing. The three tubes were transported to the laboratory as soon as possible. Since November 2010, we recorded the time of sample collection and incubation start to reduce any pre-analytical variability. All tubes were incubated for 16–24 hours according to the manufacturer’s recommendation. ELISA optical density (OD) was read using a CODA microplate processor (Biorad, CA, USA). Four IFN-γ standards (4, 1, 0.25, and 0 IU/mL) in duplicate were used to create standard curves for each analysis. The OD values were then entered into the QFT Gold analysis software v 2.50.4. The software gave test results automatically according to the manufacturer’s specifications. The nil response (Nil) was the concentration of IFN-γ in plasma from unstimulated blood. The mitogen response was calculated from the concentration of IFN-γ in plasma from PHA stimulated blood minus Nil. The concentration of IFN-γ in plasma from blood stimulated by a single cocktail of peptides representing ESAT-6, CFP-100, and part of TB7.7 minus Nil was the TB response.

### Determination of indeterminate results

We interpreted the test results according to both the results of the analysis software and quality control. QFT-GIT was considered positive if nil response was ≤ 8.0 IU/mL and TB response was ≥0.35 IU/mL and ≥25% of nil value. The result was considered negative when the TB response was <0.35 IU/mL or ≥0.35 IU/mL and <25% of nil value in case of nil response ≤ 8.0 IU/ml and mitogen response ≥0.5 IU/mL. The test was regarded indeterminate when there was a low mitogen response <0.5 IU/mL and high nil response >8.0 IU/mL, as specified by the manufacturer’s criteria. Positive and negative results of QFT-GIT were combined into one category termed as “determinate”.

### Statistical analysis

Data are expressed as medians and ranges or numbers (percentages) and mean±standard deviation. Categorical variables were compared using the Pearson's χ2 test or Fisher's exact test. The continuous variables were compared between the determinate and indeterminate group’s results using the Student t test after checking normal distribution. The likelihood ratio of indeterminate result according to quartile group of total WBC, neutrophil count, lymphocyte count, and NLR was obtained. The receiver operating characteristics (ROC) curves were constructed to evaluate the relationship between WBC parameters and indeterminate results. The correlation coefficient between NLR and age, mitogen response, nil response was calculated. The Kruskal-Wallis test was used to compare the difference of age, nil response and mitogen response in the NLR quartile groups. All variables influencing indeterminate QFT-GIT results and NLR were evaluated by multiple regression analysis. All p values less than 0.05 were considered to be statistically significant. Data analysis was carried out using MedCalc Software (ver. 13.2.0, MedCalc Software, Mariakerke, Belgium).

## Results

### Characteristics of study subjects

A total of 3,183 QFT-GIT tests were recruited during the period investigated. Of these, 410 tests were excluded because they did not have an available CBC with differential count. Two hundred seventy-eight subjects had a repetitive QFT-GIT assay. Each result of the QFT-GIT had a separate CBC with differential count, so we analyzed 2773 results from 2495 subjects. Among the 2,549 determinate QFT-GIT test results (91.9%), there were 1,165 positive (42.0%) and 1,384 negative (49.9%) results. Indeterminate QFT-GIT test results were 224 (8.1%). The majority of indeterminate results (210, 93.7%) were caused by an inadequate IFN-γ response in the PHA and 14 (6.3%) yielded a high IFN-γ response to the no antigen (Nil) (>8.0 IU/mL). There was no difference in gender, but higher age was observed in the indeterminate result group. There was a statistically significant difference in the IFN-γ response to nil, TB antigen, and mitogen between the determinate and indeterminate result groups. CBC results including total WBC, neutrophil, lymphocyte, and NLR also showed significantly different values in the determinate and indeterminate result group (Table 1).

**Table 1:** Summary of 2773 QuantiFERON-TB Gold In-Tube (QFT-GIT) tests.

Characteristic	Total	Determinate	Indeterminate	
	n = 2773	n = 2549 (91.9%)	n = 224 (8.1%)	
Age, y	50.4±20.8	50.0±20.8	55.6±20.4	p = 0.0001
Male sex, n (%)	1532 (55.2)	1407(55.2)	125(55.8)	P = 0.92
Interferon-γ response (IU/mL)				
Nil	0.33±0.93	0.28±0.63	0.84±2.42	p = 0.0006
TB antigen	1.89±3.37	2.07±3.42	-0.20±1.54	p<0.0001
Mitogen (phytohemagglutinin)	7.7±3.7	8.36±3.06	0.29±1.78	p<0.0001
CBC				
WBC, total (per μL)	8133.6±5516.7	7756.9±4281.3	12420.1±12198.5	p<0.0001
Neutrophil count (per μL)	5540.4±4911.7	5160±3654.3	9861.6±11264.8	p<0.0001
Neutrophil differential count (%)	64.1±15.8	63.2±15.2	74.2±18.2	p<0.0001
Lymphocyte count (per μL)	1722.7±1260.1	1742.3±1227.2	1500.5±1574.1	p = 0.026
Lymphocyte differential count (%)	24.5±13.6	25.2±13.2	16.4±15.2	p<0.0001
Neutrophil-to-lymphocyte ratio	4.6±6.8	4.1±5.6	10.4±13.1	p<0.0001

Data are presented as mean±standard deviation. IFN-γ response of nil indicates IFN-γ in plasma from unstimulated blood. IFN-γ response of TB antigen and mitogen are calculated IFN-γ in plasma from TB peptide stimulated minus nil and plasma from PHA stimulated minus nil, respectively. Positive and negative results of QFT-GIT are considered as “determinate”.

### Indeterminate results and lymphocyte and NLR

**Fig 1:**
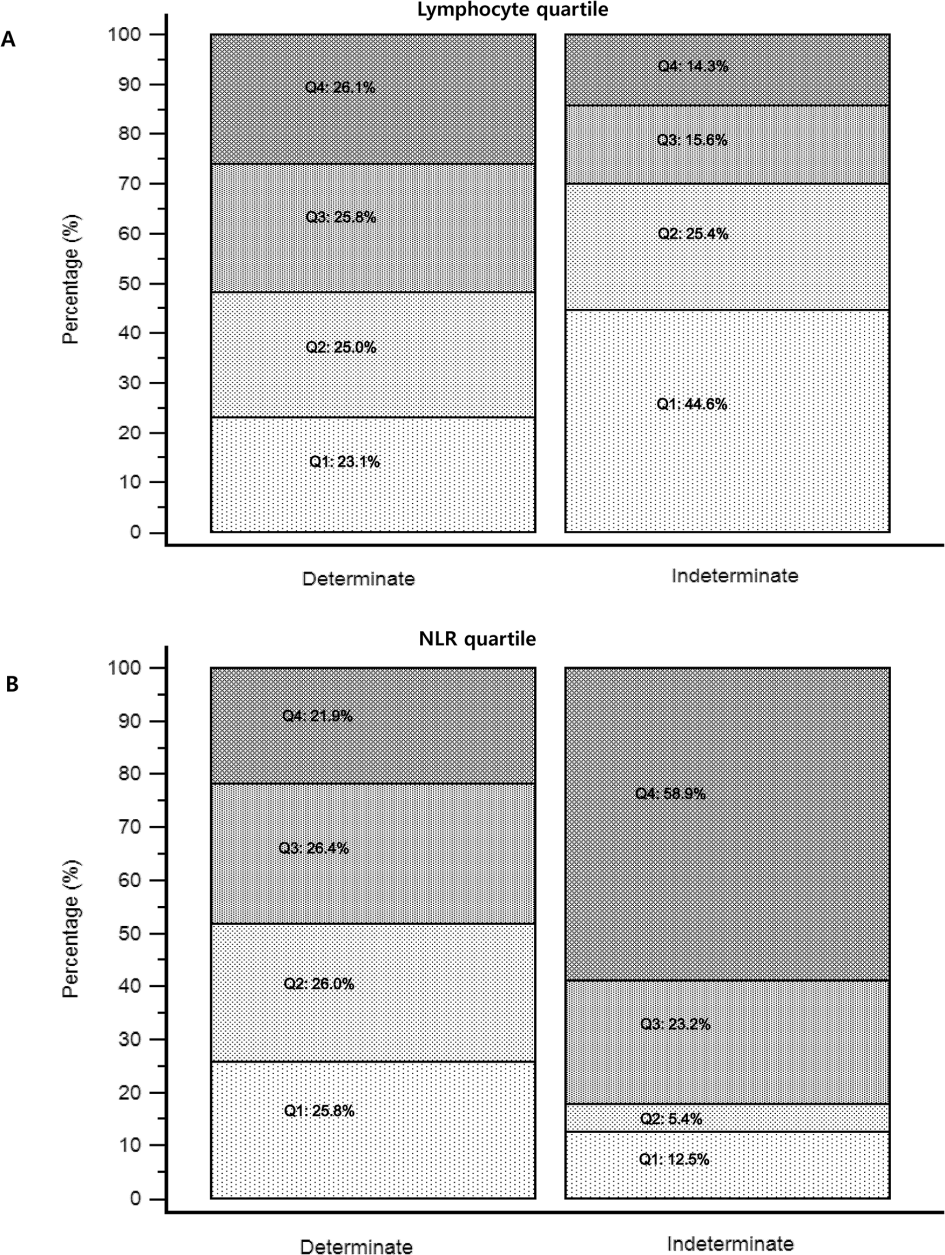
Distribution of lymphocyte quartiles and neutrophil-to-lymphocyte ratio (NLR) quartiles in determinate and indeterminate QuantiFERON-TB Gold In-Tube (QFT-GIT) assay results (n = 2773). (A) Lymphocyte quartiles (per μL) (p<0.0001 by Chi-squared test) Q1;≤1050 Q2;1051–1510 Q3;1511–2093 Q4;≥2094 (B) NLR quartiles (p<0.0001 by Chi-squared test) Q1;≤1.7 Q2;1.71–2.84 Q3;2.85–5.17 Q4;≥5.18. Positive and negative results of QFT-GIT are considered as “determinate”.

**Fig 2:**
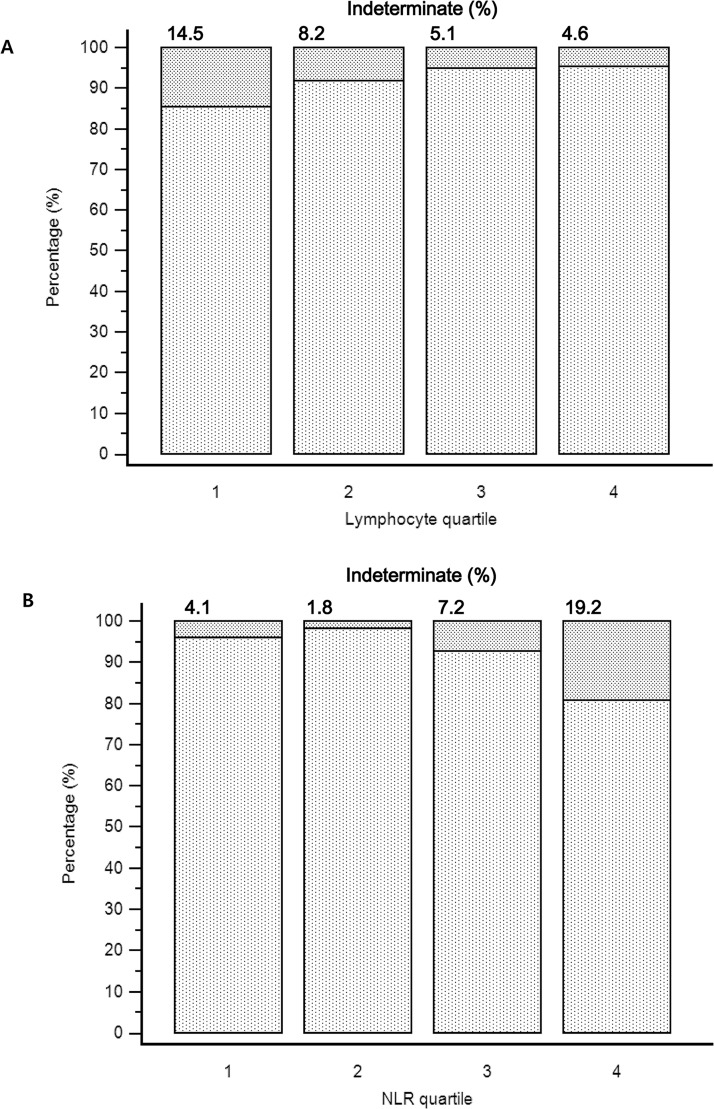
Proportions of indeterminate QuantiFERON-TB Gold In-Tube assays (n = 2773) (A) Lymphocyte quartiles (per μL). Q1;≤1050 Q2;1051–1510 Q3;1511–2093 Q4;≥2094. (B) neutrophil-to-lymphocyte ratio (NLR) quartiles. Q1;≤1.7 Q2;1.71–2.84 Q3;2.85–5.17 Q4;≥5.18.

We tried to determine whether there was any difference between determinate and indeterminate results particularly focusing on the lymphocyte and NLR. The distribution of lymphocyte quartile and NLR quartile in determinate and indeterminate QFT-GIT assay results are presented in Fig 1. For the lymphocyte quartile, 44.6% (n = 100) of 224 indeterminate results belonged to the first quartile (≤1050/ μL). There were 132 indeterminate results (58.9%) in the NLR≥5.18 group. The proportion of indeterminate results among lymphocyte quartile and NLR quartile was significantly different according to quartile (both p<0.0001). In first lymphocyte quartile (≤1050/ μL), 14.5% (100/689) showed indeterminate results and the percentage of indeterminate results was gradually decreased with increasing quartile. A marked disproportion of indeterminate results was observed in the NLR quartiles. Of the total 674 results in NLR second quartile (1.71–2.84), 12 (1.8%) showed an indeterminate result, while 19.2% (132/689) had an indeterminate result in the NLR≥5.18 group (Fig 2).

### Predictors of indeterminate results

**Fig 3:**
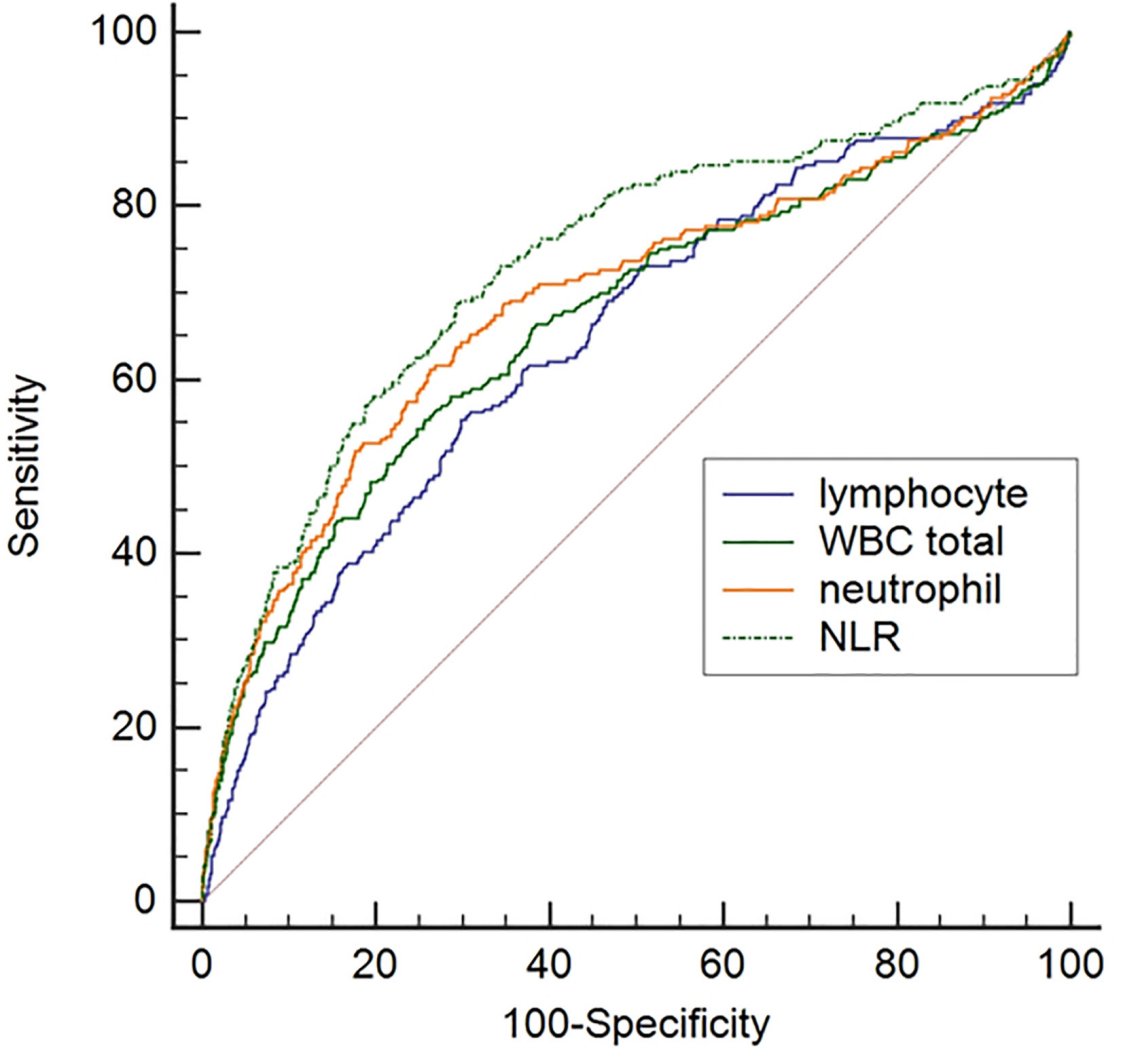
Comparison of receiver operating characteristic (ROC) curves for expecting indeterminate results in 2773 QuantiFERON-TB Gold In-Tube assays. The difference of area-under-the-curve (AUC) between neutrophil-to-lymphocyte ratio (NLR) (AUC;0.73) and neutrophil count (AUC;0.69), total WBC count (AUC;0.67), and lymphocyte count (AUC;0.65) was significant.

The likelihood ratio of indeterminate results varied among total WBC, neutrophil, lymphocyte count, and NLR quartiles. The highest likelihood ratio (LR) of indeterminate results was shown in the fourth NLR quartile (Q4≥5.18 LR 2.70, 95% CI, 2.36–3.08), followed by the fourth neutrophil count quartile (Q4≥6646/μL, LR 2.46, 95% CI, 2.14–2.82). The second quartile of NLR (Q2, 1.71–2.84) showed the lowest LR of the indeterminate results (LR 0.21, 95% CI, 0.12–0.36) (Table 2). When high NLR and lymphocytopenia were combined, the LR of indeterminate results was increased (2.81, 95% CI 2.30–3.45), unlike the cases with low NLR and lymphocytopenia (0.86, 95% CI 0.56–1.33) (Table 3). The receiver operating characteristic (ROC) curves for expecting indeterminate results showed the highest area-under-the-curve (AUC) in NLR (0.73 95% CI, 0.72–0.75) and the differences of AUC between NLR and WBC (p = 0.0019), lymphocyte (p<0.0001), and neutrophil count (p = 0.0043) were significant (Fig 3). The criterion of NLR for indeterminate result calculated from ROC curve was 4.18. The NLR and neutrophil count were independent predictors for indeterminate QFT-GIT results in the multiple regression analysis (p<0.0001 and p = 0.021, respectively). Lymphocyte count did not have significance in prediction of indeterminate QFT-GIT result in multivariate analysis but there was a significant association in univariate analysis (p = 0.0059) (Table 4).

**Table 2:** Likelihood ratio for indeterminate results of 2773 QuantiFERON-TB Gold In-Tube (QFT-GIT) tests based on the variables.

	Determinate	Indeterminate	Likelihood ratio
	(n)	(n)	(95% CI)
Total WBC count (per μL)			
Q1 (≤5349)	654	38	0.66 (0.49–0.89)
Q2 (5350–7030)	665	26	0.45 (0.31–0.64)
Q3 (7031–9582)	649	45	0.79 (0.60–1.03)
Q4 (≥9583)	581	115	2.25 (1.95–2.61)
Lymphocyte count (per μL)			
Q1 (≤1050)	589	100	1.93 (1.64–2.27)
Q2 (1051–1510)	638	57	1.02 (0.81–1.29)
Q3 (1511–2093)	657	35	0.61 (0.44–0.83)
Q4 (≥2094)	665	32	0.55 (0.40–0.76)
Neutrophil count (per μL)			
Q1 (≤2979)	655	37	0.64 (0.48–0.87)
Q2 (2980–4396)	672	24	0.41 (0.28–0.60)
Q3 (4397–6645)	652	40	0.70 (0.52–0.93)
Q4 (≥6646)	570	123	2.46 (2.14–2.82)
Neutrophil-to-lymphocyte ratio			
Q1 (≤1.7)	658	28	0.48 (0.34–0.69)
Q2 (1.71–2.84)	662	12	0.21 (0.12–0.36)
Q3 (2.85–5.17)	672	52	0.88 (0.69–1.13)
Q4 (≥5.18)	557	132	2.70 (2.36–3.08)

Positive and negative results of QFT-GIT are considered as “determinate”.

**Table 3:** Likelihood ratio for indeterminate results based on the combination of lymphocyte count and NLR in 2773 QuantiFERON-TB Gold In-Tube (QFT-GIT) tests.

	Determinate	Indeterminate	Likelihood ratio
	(n)	(n)	(95% CI)
NLR (≥5.18) and Lymphocyte (≤1050/μL)	324	80	2.81 (2.30–3.45)
NLR (≥5.18) and Lymphocyte (≥1051/μL)	233	52	2.54 (1.95–3.32)
NLR (≤5.17) and Lymphocyte (≤1050/μL)	265	20	0.86 (0.56–1.33)
NLR (≤5.17) and Lymphocyte (≥1051/μL)	1729	72	0.47 (0.39–0.58)

Positive and negative results of QFT-GIT are considered as “determinate”.

**Table 4:** Multiple regression analysis between indeterminate QuantiFERON-TB Gold In-Tube result and variables (n = 2773).

	Correlation coefficient	r_partial_	t	p
Age	0.073	0.023	1.234	0.217
Neutrophil-to-lymphocyte ratio	0.254	0.112	5.927	<0.0001
WBC total	0.23	-0.012	-0.665	0.505
Lymphocyte count	-0.052	0.007	0.399	0.689
Neutrophil count	0.26	0.043	2.297	0.021

### Neutrophil-to-lymphocyte ratio

**Fig 4:**
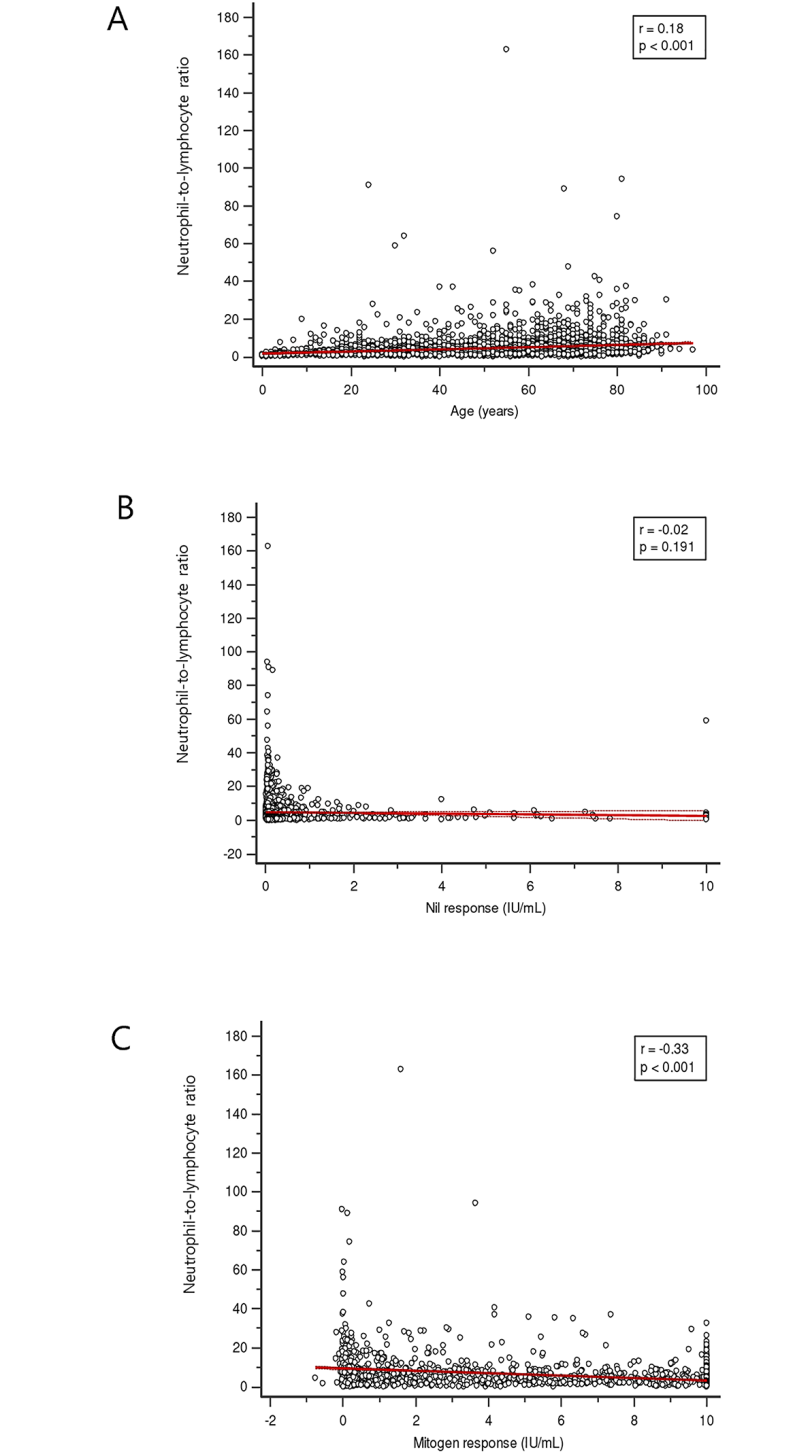
Correlation between the neutrophil-to-lymphocyte ratio (NLR) and variables. (n = 2773) (A) Age. The dotted lines indicate the 95% confidence interval. (B) Nil response in QuantiFERON-TB Gold In-Tube (QFT-GIT) assay. Nil response indicates the concentration of interferon-γ in plasma from unstimulated blood. (C) Mitogen response in QFT-GIT assay. Mitogen response is calculated interferon-γ in plasma from phytohemagglutinin stimulated minus nil. The NLR shows a weak positive correlation with age and a weak negative correlation with mitogen response.

**Fig 5:**
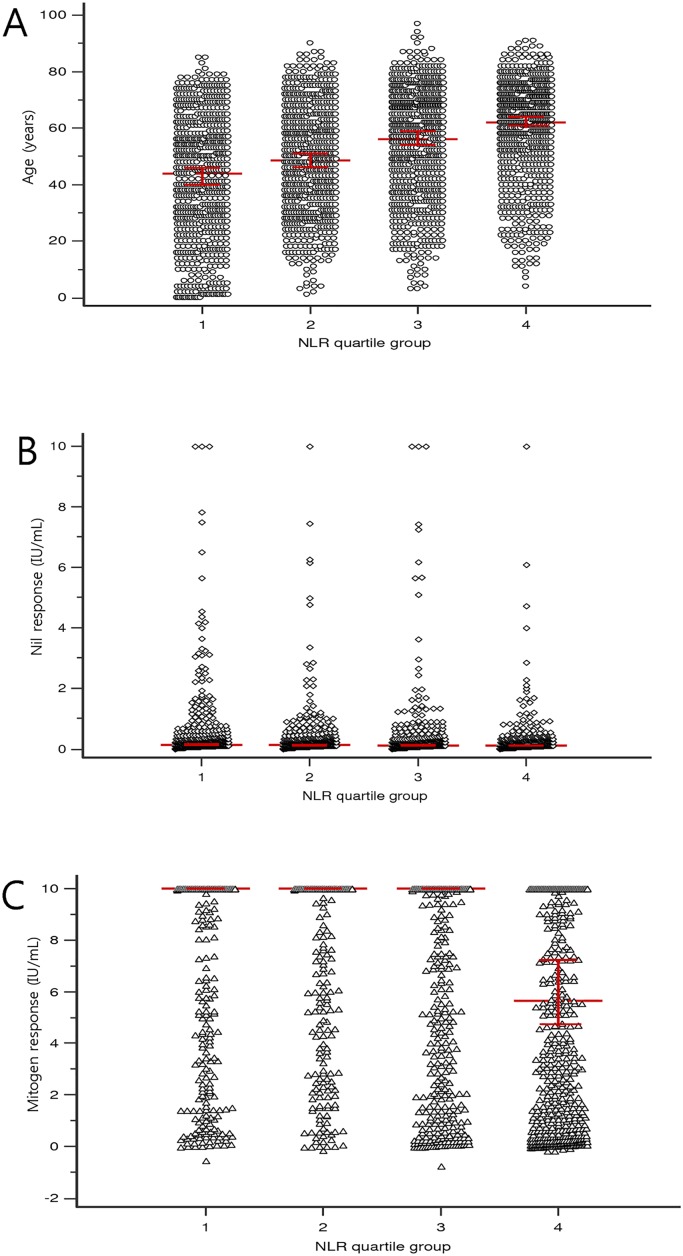
Relationship between variables and neutrophil-to-lymphocyte ratio (NLR) quartile group (n = 2773). (A) Age NLR quartile Q1;≤1.7 Q2;1.71–2.84 Q3;2.85–5.17 Q4;≥5.18. The red lines indicate the median and 95% confidence interval. (B) Nil response according to NLR quartile group in QuantiFERON-TB Gold In-Tube (QFT-GIT) assay. Nil response indicates the concentration of interferon-γ in plasma from unstimulated blood. (C) Mitogen response according to NLR quartile group in QFT-GIT assay. Mitogen response is calculated interferon-γ in plasma from phytohemagglutinin stimulated minus nil.

As the NLR was a significant predictor for indeterminate QFT-GIT results in multiple regression analysis, we additionally analyzed the relationship between NLR and other parameters. The NLR of male subjects was significantly higher (median, 3.10; interquartile range (IQR), 1.79–5.63; mean± SD, 4.88±5.98) than that of female (median, 2.63; IQR, 1.62–4.67; mean± SD, 4.36±7.63) (p = 0.043). For univariate analysis, age and NLR were positively correlated (r = 0.18, p<0.001). Nil response did not have a significant association with NLR (r = -0.02, p = 0.191) however, the mitogen response to PHA was negatively associated with NLR (r = -0.33, p<0.001) (Fig 4). In multiple regression analysis, WBC total, neutrophil count, lymphocyte count, and mitogen response were independent predictor of NLR. The lymphocyte count was the highest independent predictor for NLR (t-value = -11.9, p<0.0001). Age and nil response showed no significance of relationship with NLR in multiple regression analysis (Table 5). The age, nil response and mitogen response according to NLR quartile are shown in Fig 5. The median of age gradually increased with NLR quartile and median of mitogen response among NLR quartile showed an extreme asymmetry. Median of mitogen response in fourth NLR quartile was 5.59 IU/mL (95% CI, 4.59–7.21).

**Table 5:** Multiple regression analysis between neutrophil-to-lymphocyte ratio and variables (n = 2773).

	Correlation coefficient	r_partial_	t	p
Age	0.176	0.015	0.838	0.402
WBC total	0.498	0.042	2.221	0.026
Neutrophil count	0.608	0.098	5.201	<0.0001
Lymphocyte count	-0.288	-0.221	-11.924	<0.0001
Mitogen response^a)^	-0.322	-0.164	-8.749	<0.0001
Nil response ^a)^	-0.023	-0.004	-0.227	0.82

^a)^ Mitogen and nil response were calculated from QuantiFERON-TB Gold In-Tube assay.

## Discussion

Our study showed that NLR is an independent predictor of indeterminate QFT-GIT results. Additionally, neutrophil count showed significance in the prediction of indeterminate results. Lymphocyte count grouped by quartile showed an increased likelihood ratio for indeterminate results but the lymphocyte count did not have significance in multivariate analysis. The variables influencing NLR were gender, total WBC, neutrophil count, lymphocyte count, and mitogen response to PHA. Age was significantly associated with NLR in univariate analysis, but there was a lack of significance in multiple regression analysis.

Indeterminate QFT-GIT assay results delay clinical management because they do not represent the *M*. *tuberculosis* infection status of the patient. Therefore, several studies have been performed to evaluate the predictors for indeterminate QFT-GIT results. The predictors have varied according to characteristics of studied patients and the statistical method used. In 631 patients with rheumatic diseases, there were almost eleven clinical, laboratory, and technical predictors for indeterminate QFT-GIT results in univariate analysis. After multivariate analysis, systemic lupus erythematosus, use of sulfasalazine or a tumor necrosis factor inhibitor, and a manual ELISA system remained significantly independent predictors of indeterminate results [12]. In studies of a heterogeneous patient population, immunosuppressive condition, hypoalbuminemia, and low hemoglobin level were mainly associated with increased indeterminate QFT-GIT results [14,16,19]. Regarding lymphocytopenia, the association with the frequency of indeterminate results was not revealed after multivariate logistic regression analysis [12,16]. These findings are consistent with our study. In univariate analysis, the lymphocyte count showed a significant association with indeterminate results, but the significance was not sustained in multivariate analysis.

To our knowledge, this is the first study to show the predicting value of NLR on indeterminate QFT-GIT results. Our study indicates almost one-fifth of QFT-GIT results were indeterminate in the increased NLR quartile (≥5.18). The majority of indeterminate results were caused by an inadequate IFN-γ response after PHA stimulation. In our study, 93.7% (210/224) were included in this category and 95.8% (114/119) were recently reported [21]. This indicate that patients with high NLR might have enhanced a possibility of low IFN-γ secretion in response to PHA. Recently, a subset of neutrophils in patients with severe injuries has been shown to inhibit T cell responses through the integrin Mac-1. The presence of Mac-1–induced suppression was demonstrated by an increase in IFN-γ production induced by PHA upon addition of the blocking antibody 44a [22]. The inhibitory effect on T cells by neutrophils could explain low IFN-γ secretion in response to PHA in patients with high NLR. The relationship between high NLR and inadequate IFN-γ response to PHA could be extended to the prognostic role of NLR in various disease categories [1,3,5,6]. Indeterminate QFT-GIT results have shown prognostic value for serious clinical events in HIV-1-infected patients with advanced quantitative CD4 T cell depletion [15]. Although there was no depiction of NLR in that study, the loss of global T cell function may suggest the role of neutrophil in agreement with our result.

In addition, neutrophils have been considered as effectors in both the innate and adaptive immunoregulatory networks and were found to be related in physiological and pathological conditions beyond the immune system, such as in autoimmunity, cancer, and atherosclerosis [23,24]. Our result of markedly disproportionate indeterminate results according to NLR quartile indicates the importance of a numerical balance between neutrophils and lymphocytes. In studies of a healthy population, the mean level of NLR was reported as 1.77±0.4 in 40 to 49 years of age and 2.34±1.07 in 759 healthy control [25,26]. These values are concordant with our second NLR quartile (1.71–2.84) that had the lowest indeterminate rate (1.8%). The normal NLR may have a protective effect against inadequate IFN-γ response after PHA stimulation. This phenomenon was not observed in the lymphocyte quartiles. The simple numeric value of NLR implies the importance of balance between cellular compartments.

High NLR has been attributed to neutrophilia considered as an indicator of inflammation. In a large population, patients with chronic inflammatory conditions have been more likely to have indeterminate QFT-GIT results compared to healthy subjects. Also, among patients with chronic inflammatory conditions, only glucocorticoid use was associated with an increased relative risk of indeterminate results [27]. Glucocorticoid is well-known to induce leukocytosis [28,29]. These findings support the association between high NLR and indeterminate results in this study.

Aging has known to be associated with NLR level, and the elder age group possessed higher NLR [25]. In this study, there was a positive correlation between age and NLR in univariate analysis. The NLR was significantly elevated in patients with Alzheimer's disease compared to healthy controls, but after adjustment for age, sex, and *APOEε4* allele status, there was no significance between NLR and Alzheimer's disease [26]. That finding is consistent with our result that age failed to show significance of relationship with NLR under the multiple analysis. It is necessary to find the precise relationship between age and NLR in the aspect of immune response.

The cut-off level of NLR for prediction of adverse events has been considered to be from 4.5 to 5.0 in various diseases. The adjusted hazard ratio of NLR >4.5 for coronary heart disease deaths was 2.68 (95% CI, 1.07–6.72) [6] and a threshold of NLR >5 was the most consistently used in patients with cancer [2]. The criterion of NLR for indeterminate result calculated from ROC curve was 4.18 and the likelihood ratio of NLR ≥5.18 for indeterminate QFT-GIT result was 2.70 (95% CI, 2.36–3.08) in this study. In terms of the threshold of NLR for increased risk in pathologic conditions, there was a quite narrow range, from 4.5 to 5.2. More simplified measure of NLR indicating inflammatory response could be derived from a prospective study in large scaled cohort.

This study had some limitations. Only demographic and CBC data, rather than clinical information of individual patient could be used. Although the difference of AUC between NLR and WBC, lymphocyte, and neutrophil count for expecting indeterminate results was significant, the AUC of NLR was not high (0.73). This indicates the relationship between NLR and indeterminate results may be weak. The underlying diseases or bedridden state have been an important factor for indeterminate results and immunosuppressive drugs have an influence on indeterminate results. We analyzed the lymphocyte, neutrophil, and total WBC, but other laboratory parameters, such as hemoglobin and albumin, were not included. The hypoalbuminemia has showed significantly high odds ratio for indeterminate results and is related to inflammation [16,30]. The high NLR has been associated with hypoalbuminemia in patients with lung cancer [31]. Second, this study was conducted retrospectively so some QFT-GIT results were not included because of unavailable CBC data. Furthermore, we monitored the incubation delay by recording the time of sample collection, but the technical factors of indeterminate QFT-GIT results were not considered in this study.

In summary, we revealed that the NLR is an independent predictor of indeterminate QFT-GIT results. Because the majority of indeterminate results were caused by inadequate IFN-γ response after PHA stimulation, a high NLR would reflect T cell inhibition by neutrophils. Low frequency of indeterminate results in group with normal NLR may imply the importance of a balance between the two cellular compartments in physiological and pathological conditions.
